# Ecological and genetic relationships of the *Forest-M *form among chromosomal and molecular forms of the malaria vector *Anopheles gambiae sensu stricto*

**DOI:** 10.1186/1475-2875-8-75

**Published:** 2009-04-21

**Authors:** Yoosook Lee, Anthony J Cornel, Claudio R Meneses, Abdrahamane Fofana, Aurélie G Andrianarivo, Rory D McAbee, Etienne Fondjo, Sekou F Traoré, Gregory C Lanzaro

**Affiliations:** 1 School of Veterinary Medicine, Department of Pathology, Microbiology and Immunology, University of California – Davis, Davis, CA, USA; 2 Department of Entomology, University of California – Davis, Davis, CA, USA; 3 Malaria Research and Training Center, Faculty of Medicine, University of Mali, Bamako, Mali; 4 National Malaria Program, Ministry of Health, Yaoundé, Cameroon

## Abstract

**Background:**

*Anopheles gambiae sensu stricto*, one of the principal vectors of malaria, has been divided into two subspecific groups, known as the M and S molecular forms. Recent studies suggest that the M form found in Cameroon is genetically distinct from the M form found in Mali and elsewhere in West Africa, suggesting further subdivision within that form.

**Methods:**

Chromosomal, microsatellite and geographic/ecological evidence are synthesized to identify sources of genetic polymorphism among chromosomal and molecular forms of the malaria vector *Anopheles gambiae s.s*.

**Results:**

Cytogenetically the Forest M form is characterized as carrying the standard chromosome arrangement for six major chromosomal inversions, namely 2La, 2Rj, 2Rb, 2Rc, 2Rd, and 2Ru. Bayesian clustering analysis based on molecular form and chromosome inversion polymorphisms as well as microsatellites describe the Forest M form as a distinct population relative to the West African M form (Mopti-M form) and the S form. The Forest-M form was the most highly diverged of the *An. gambiae s.s*. groups based on microsatellite markers. The prevalence of the Forest M form was highly correlated with precipitation, suggesting that this form prefers much wetter environments than the Mopti-M form.

**Conclusion:**

Chromosome inversions, microsatellite allele frequencies and habitat preference all indicate that the Forest M form of *An. gambiae *is genetically distinct from the other recognized forms within the taxon *Anopheles gambiae sensu stricto*. Since this study covers limited regions of Cameroon, the possibility of gene flow between the Forest-M form and Mopti-M form cannot be rejected. However, association studies of important phenotypes, such as insecticide resistance and refractoriness against malaria parasites, should take into consideration this complex population structure.

## Background

*Anopheles gambiae sensu stricto *is one of the major vectors responsible for malaria transmission in Africa. Genetic polymorphism in *An. gambiae s.s*. is a major factor contributing to the widespread occurrence of this disease vector both in time and space. Population genetic analysis of *An. gambiae s.s*. populations across Africa has revealed at least three genetically distinct groups within this single species[1]. However, an extensive literature describes a highly complex population genetic structure on a much smaller spatial scale in West and Central Africa [2-8].

Touré and his colleagues [5] examined the distribution of five paracentric chromosome inversions on the right arm of chromosome 2 (2R j, b, c, d and u). They established a convention for describing the karyotype of an individual mosquito, wherein a dash ('-') is used to designate the standard homozygote, '1' for a heterozygote and '2' for the inverted homozygote for each chromosome inversion. Each karyotype, thus, includes five characters, each character denoting the "genotype" for each of the five 2R chromosome regions that include the j, b, c, d and u inversions. Analysis of karyotype frequencies among *An. gambiae s.s*. collected from a number of villages led to the identification of discrete subpopulations within this species which can be distinguished by their karyotype. These subpopulations were given non-Linnean designations and are collectively known as "chromosomal forms" [2,5]. Five chromosomal forms have been identified and named *Mopti*, *Bamako*, *Bissau*, *Forest *and *Savanna *according to the regions from which they were first collected, underscoring the association of each with a particular type of habitat. Various field-based studies clearly demonstrate that these forms show distinct patterns of seasonal and geographic distributions [2,5,6,9]. Detailed analysis of populations where multiple forms exist sympatrically revealed a high degree of reproductive isolation between the forms, with a strong preference for mating within rather than between forms [8].

Attempts to develop molecular diagnostics for the chromosomal forms culminated in the recognition of two distinct sequences in the intergenic spacer region of the ribosomal DNA locus [10,11]. A PCR-RFLP technique is now widely used to distinguish between individuals carrying one or the other of the ribosomal "alleles". These have been termed "molecular forms" of *An. gambiae*. There are two molecular forms, M and S, which in some places (e.g. Mali) nearly always correspond with the chromosomal forms (S = *Savanna *or *Bamako *Form, M = *Mopti *Form). In many places, however, the association between chromosomal form and molecular form appears to break down. For example in Senegal the *Savanna *chromosomal form is frequently of the M molecular form [12,13] and in Cameroon populations of the *Forest *chromosomal form may be either M or S [14].

Analysis of gene flow between molecular forms has revealed that, as with the chromosomal forms, there is very strong positive assortative mating within molecular forms with little or no mating between forms [8,14]. Wondji *et al*. [14] found significant genetic differentiation between the M and S forms in Cameroon. The high levels of differentiation (F_ST_) they observed were uniformly distributed over ten microsatellite loci covering the entire genome, leading them to conclude that differentiation is the consequence of complete reproductive isolation between the M and S forms in Cameroon. Likewise Slotman *et al*. [15] reported significant genetic differentiation between the M and S forms in Cameroon. They also compared M form populations in Cameroon with those in Mali and surprisingly found even higher levels of divergence than those reported between the M and S forms in both countries. These findings led them to conclude that there are two distinct M forms, which they termed Mopti-M (described from Mali) and Forest-M (described from Cameroon). These findings led to the hypothesis that both reproductive isolation, as determined by molecular form, and ecological factors affect the genetic makeup of *An. gambiae s.s*. and other markers such as chromosomal inversions should reflect such genetic divergence.

On the contrary, Yawson *et al*. [16] report a lack of differentiation between M and S forms in Ghana and Burkina Faso. They used both Bayesian clustering methods and F_ST_-based analysis of allele frequencies at seven microsatellite loci. Although they did not find M/S hybrids, an admixture analysis suggests introgression between the two forms. They did, however, find strong differentiation between populations occupying different ecological zones, irrespective of M or S form, leading them to conclude that ecological barriers are more significant in restricting gene flow among populations than is reproductive isolation between the two forms.

In this paper, the results of a study investigating associations between chromosome inversions and molecular forms as well as the association between chromosome inversions and levels of precipitation are presented.

## Methods

### Sample collection

**Figure 1:**
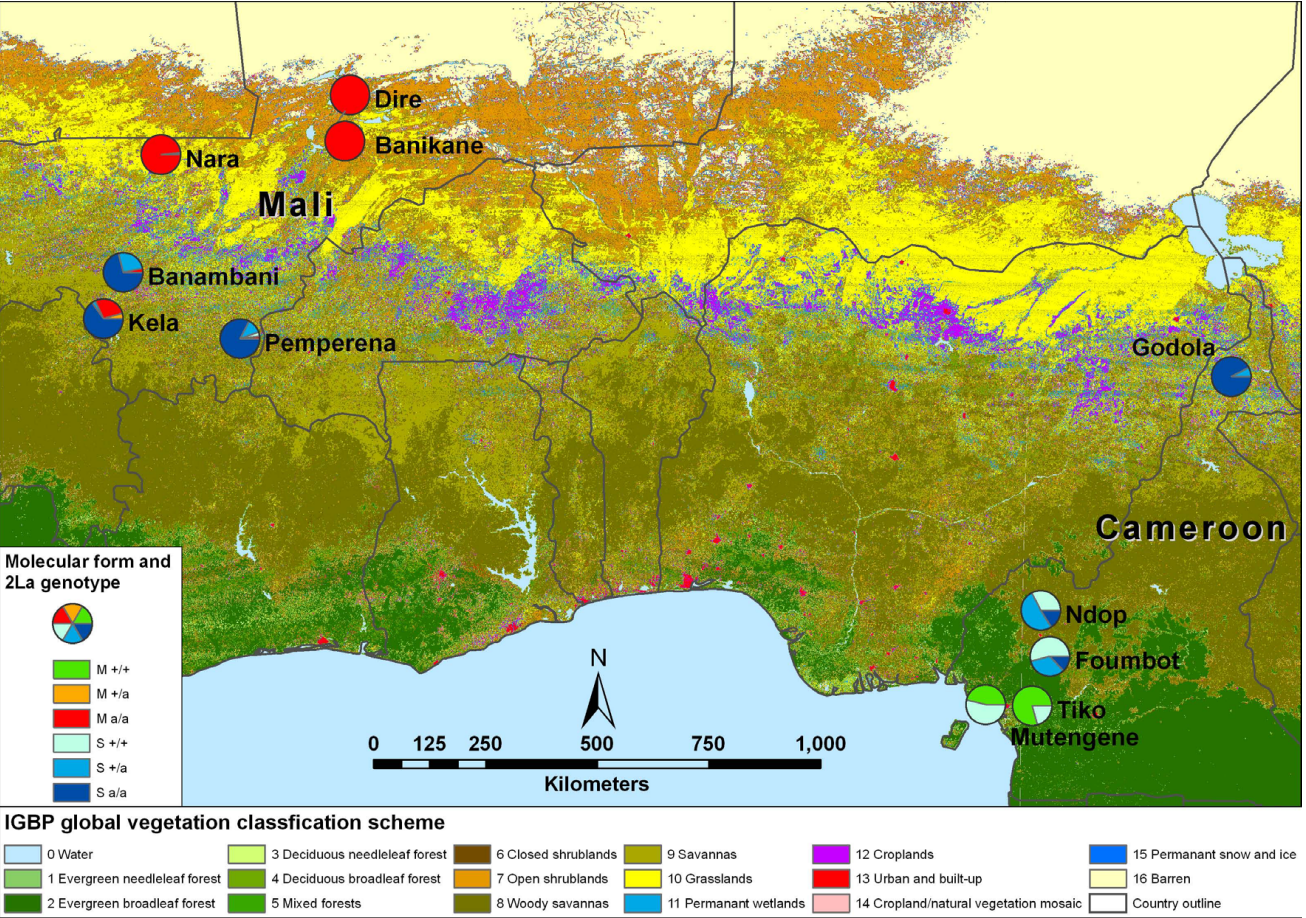
**Map of collection sites and genotype distributions**. Relative proportion of molecular form and 2La chromosome inversion karyotypes for collection sites in Mali and Cameroon are shown in pie charts. Colours and corresponding land cover types for all 17 International Geosphere-Biosphere Programme (IGBP) global vegetation classes are shown in the legend at the bottom of the figure.

Adult females of *An. gambiae s.s*. were collected indoors using aspirators between 2002 and 2006 from the locations shown in Figure 1. Sample sizes range from 13 to 157 per site (Table 1). Mutengene is reported as Mutenguene and Pemperena as Pimperena in other publications. This paper followed the names listed in the worldwide index of cities and towns, Global Gazetteer version 2.1 (Falling Rain Genomics, Palo Alto, CA). Collections were made during the rainy season (collection dates are provided in Table 1). Ovaries were removed from semi-gravid females and stored in Carnoy's solution for cytogenetic analysis of polytene chromosomes [17]. Carcasses were stored in alcohol for subsequent DNA extraction. Genomic DNA was extracted using a DNeasy^® ^extraction kit (Qiagen, Valencia, CA, USA).

**Table 1:** Average precipitation in millimeters recorded near collection sites.

**site latitude**		**longitude**	**Major IGBP land cover type within 10 km radius**	**Mean annual precipitation (mm/year)**	**Collection Date**
**Cameroon**					

Tiko	4.0786	9.3681	Evergreen broadleaf forests	2789 ± 1207	Sep. 2003 Aug. 2006

Mutengene	4.0994	9.3081	Evergreen broadleaf forests	2800 ± 1240	Oct. 2003

Foumbot	5.4851	10.6000	Woody savannas	1774 ± 280.3	Jul. 2004 Aug. 2006

Ndop	6.0000	10.4167	Woody savannas	1749 ± 271.0	Aug. 2004

Godola	10.7000	14.2500	Mosaic of grasslands, permanent wetlands and croplands	826.8 ± 256.6	Aug. 2005

**Mali**					

Pemperena	11.4670	-5.7000	Mosaic of woody savannas and savannas	1004 ± 133.2	Aug. 2002

Kela	11.8870	-8.4474	Savannas	958.5 ± 105.0	Oct. 2006

Banambani	12.8000	-8.0500	Savannas	830.9 ± 157.5	Jul. 2005 Aug. 2006

Nara	15.1630	-7.2917	Grasslands	380.1 ± 98.16	Nov. 2003 Jul. 2005

Banikane	16.0400	-3.5900	Open shrublands	141.5 ± 69.82	Nov. 2002 Oct. 2003

Dire	16.3670	-3.4833	Open shrublands	170.8 ± 72.77	Nov. 2002

### Species and molecular form diagnostic

The PCR assay described by Scott *et al*. [18] was used to identify members of the *An. gambiae *species complex. Molecular forms within *An. gambiae s.s*. were identified based on published methods [10,19].

### Cytogenetic analysis

Polytene chromosomes were extracted from ovarian nurse cells using the protocol described by Hunt [17]. Chromosome banding patterns were examined using an Olympus BX-50 phase contrast microscope. Species identification and karyotype scoring were accomplished using the polytene chromosome map for the *An. gambiae *complex developed by Coluzzi *et al*. [20] (maps available as supplement material). The chromosomal forms of *An. gambiae s.s*. were distinguished by scoring paracentric inversions on the right arm of chromosome 2, as described by Coluzzi *et al*. [9]. The genotypes of six chromosome inversions --- 2La, 2Rj, 2Rb, 2Rc, 2Rd, and 2Ru --- were scored for each individual mosquito. Photographic images of chromosomes for the majority of individual mosquitoes used in this study are available on request.

### Microsatellite genotyping

Twenty microsatellite loci located on the second and the third chromosome were screened, namely AG2H57, AG2H85, AG2H125, AG2H135, AG2H164, AG2H175, AG2H197, AG2H675, AG3H119, AG3H127, AG3H242, AG3H249, AG3H312, AG3H555, AG3H577, AG3H59, AG3H746, AG3H812, AG3H817, AG3H93 [21]. The same set of 12 markers on the third chromosome was used in the previous study by Slotman *et al*. [15]. Some specimens used in Slotman *et al*. [15] that had associated karyotype data are also included in this study. Microsatellite markers were amplified using fluorescent primers and a PTC-225 thermolcycler (MJ Research, Watertown, MA). PCR products were mixed with a DS-30 Matrix Standard Kit (Dye Set D) (Applied Biosystems, Foster City, CA) and run on an ABI PRISM^® ^3100 Genetic Analyzer. Electrophoretograms were analysed using ABI PRISM^® ^GeneScan analysis software and Genotyper 3.7 NT.

### Environmental data

Average annual precipitation for each collection site listed in Table 1 were computed using daily precipitation data, over multiple years, from weather stations within 15 km radius of each sample collection site. Daily precipitation recorded in Cameroon stations near mosquito collection sites was acquired by personal communication with Dr. Yongkang Xue in the Department of Geography at the University of California – Los Angeles. Precipitation data for Mali stations were acquired by personal communication with Dr. Charles E. Taylor in the Department of Ecology and Evolutionary Biology at the University of California – Los Angeles. These data are provided in Additional file 1.

The International Geosphere-Biosphere Programme (IGBP) global vegetation classification scheme in 1 km resolution collected in 2004 was acquired in the form of the Land Cover Yearly L3 Global 1 km (MOD12Q1) data set produced by the NASA Moderate Resolution Imaging Spectroradiometer (MODIS) project [22,23]. The data is in Hierarchical Data Format (HDF), which is the standard data format for all NASA Earth Observing System (EOS) data products. The HEG version 2.9 (HDF-EOS to Geotiff) conversion tool was used to convert the acquired HDF file to Geotiff [24]. ArcMap version 9.1 was used to draw maps illustrating the habitat types surrounding each collection site.

### Statistics

STRUCTURE software v2.2 [25-27] was used for Bayesian clustering analysis of seven markers which include genotypes of molecular form and of 2La, 2Rj, 2Rb, 2Rc, 2Rd, and 2Ru chromosome inversions. Six consecutive symbols are used to describe karyotypes representing the genotype of inversions 2La, 2Rj, 2Rb, 2Rc, 2Rd, and 2Ru. '-' represents the standard (uninverted) homozygous arrangement, '1' heterozygotes, '2' inverted homozygotes. So, for example an individual that is homozygous for the inverted arrangement at 2La (2), homozygous standard for 2Rj (-), heterozygous for 2Rb (1), heterozygous for 2Rc (1), homozygous standard for 2Rd (-) and heterozygous for 2Ru (1) would be represented as: 2-11-1. Scoring of karyotypes that include inversions in the heterozygous condition is often ambiguous. For example, it is not possible to distinguish the 2Rbcu/+ from 2Rbc/u karyotype (i.e. gametic phase is uncertain). Regardless of gametic phase or haplotype, the input for this genotype is (- 11-1, -----) or (-1---, --1-1). For this type of Bayesian analysis, either input represents exactly the same genotype so will have the same effect on the outcome of the clustering analysis. The Bayesian model does not distinguish the two but considers the fact that the corresponding individual is heterozygous for 2Rb, 2Rc, and 2Ru. Linkage between loci is something the model explores when it tries to find the most likely solution (grouping).

The same analyses were applied to microsatellite genotypes. Individuals were clustered into a hypothetical number of discrete populations, *K *= [1,8] and the probability of the data given *K*, Prob(*X*|*K*), was computed for each run. Various combinations of burn-in period and sampling period were tested. In this case, a burn-in period of 100,000 and sampling period of 100,000 were sufficient. The probability of each individual belonging to each of the K populations was plotted using *distruct *software [28]. Raw data is provided in Table S1.

The ELB algorithm [29] as implemented in the Arlequin software package [30] was used to identify the gametic phase of karyotypes. A burn-in period of 10,000 and sampling period of 1,000 were used for estimating gametic phase. Estimated gametic phase information was subsequently used for a Hardy- Weinberg equilibrium (HWE) test. The Guo's Exact HWE Test [31] was performed both on the locus and gamete level for each collection, which consists of mosquitoes collected at the same site, same day, and of the same molecular form. A burn-in period of 100,000 and sampling period of 100,000 was used to calculate the probability of HWE. Arlequin was also used to calculate pair-wise F_ST _values based on microsatellite genotypes among the six groups identified in Bayesian clustering analysis. The neighbourjoining algorithm implemented in the *neighbor *program, a feature of *Phylip v.3.67 *[32,33] was used to illustrate relationships among groups. The *drawtree *program, also included in *Phylip v.3.67*, was used to generate a phylogenetic tree based on a pair-wise F_ST _distance matrix. *R *software [34] was used for drawing boxplots, computing Wilcoxon rank sum tests, and performing linear regression.

## Results

### Distribution of *An. gambiae s.s*. forms and inversions

The distribution of molecular forms and 2La inversion genotypes (karyotypes) are illustrated in Figure 1. In Cameroon, S forms were found in all five sample locations, while M forms were localized in the evergreen broadleaf forest region below latitude 4.5°N. In Mali, S forms were dominant below latitude 12°N in mostly savanna regions, while M forms were predominant above 15°N where land cover types are mostly grasslands or open shrublands (Table 1, Figure 1).

Chromosome inversion polymorphism was examined on the second chromosome, specifically 2La, 2Rj, 2Rb, 2Rc, 2Rd and 2Ru. Karyotypes, as well as molecular forms were distributed in a non-random fashion. For instance, only 25% of samples collected in Cameroon were 2La inversion homozytoes (a/a), mostly concentrated in a village called Godola located in northern Cameroon. The 2La inversion was completely absent in Tiko and Mutengene. In contrast, about 87% of specimens collected in Mali were 2La inversion homozygotes (Table 1, Figure 1) and only three specimens were 2La standard homozygotes (+_a_/+_a_).

The M forms found in Mali were polymorphic for all six inversions (96% of the samples from Mali carried at least one copy of the 2Rb and/or c inversions), but were predominantly 2La inversion homozygotes. M form populations in Cameroon were predominantly fixed for the standard karyotype (M------) for all six inversions, including 2La. In the savanna region of northern Cameroon the M form occurred, but was rare (e.g. 1 M form from Maroua, 10.60 N 14.33E, 3 M forms from Ourodoukoudje, 9.083 N 13.683E and 4 M forms from Raio, 9.117 N 13.717E). In these cases all had the M2-22-- (2La a/a, 2R bc/bc) karyotype. Due to very small sample size, these sites were not included in this study.

The S forms collected in Cameroon were polymorphic for the 2La and 2Rb inversions, although inversion homozygotes for 2La and 2Rb were relatively rare compared to Mali. In Cameroon, 30% (43/143) of S forms were 2Rb homozygotes, but 93% of these were from a single site (Godola). The standard arrangement for 2Rb (+_b_/+_b_) was also common, occurring at a frequency of 45% (64/143) in Cameroon. Fewer +_b_/+_b _S form individuals were observed in Mali (18%, 48/273) than in Cameroon. S form 2Rb homozygotes occurred with equivalent frequencies in the villages of Banambani, Kela and Pemperena, perhaps because these occur in similar land cover types (Table 1). In drier areas of Mali such as grasslands and open shrublands, the S form was rare. The correlation of 2La and 2Rb inversions with an environmental parameter, precipitation, is discussed below.

### Identification of discrete populations based on molecular form and karyotype

**Figure 2:**
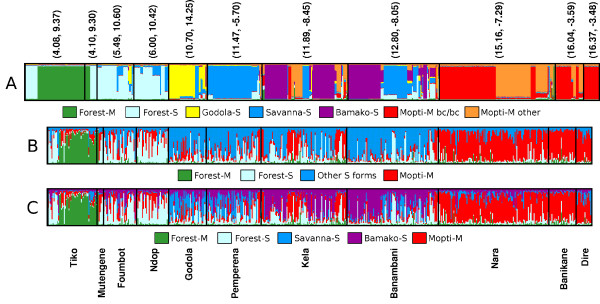
***Structure *clustering results**. A: Membership coefficients of all individuals for K = 7. Bayesian clustering based on seven markers, which include molecular form, the 2La, 2Rj, 2Rb, 2Rc, 2Rd, and 2Ru inversions. Each vertical line represents a single individual. Different colours represent different clusters (= forms). The green colour corresponds to Forest-M form, light-blue to Forest-S form, yellow to Godola-S form, blue to Savanna-S form, purple to Bamako-S form, red to Mopti-M form bc/bc homozygotes, and orange to the "other" Mopti-M forms. Individuals are ordered such that the latitude of collection sites increase from left to right. The latitude and longitude of each collection site are indicated on top of the bar plots and the names of the collection sites are indicated below the bar plots. The associated karyotypes are summarized in Table 2. B: Membership coefficients for K = 4 clustering based on microsatellite markers. The green colour corresponds to Forest-M form, light-blue to Forest-S form, Blue to all other S forms, and red for Mopti- M form. Note that separation of Godola-S, Savanna-S and Bamako-S were not supported at K = 4. C: Membership coefficient for K = 5 clustering based on microsatellite markers. The color scheme is similar to Figure 3B, with the addition of the Bamako-S form indicated in purple.

**Figure 3:**
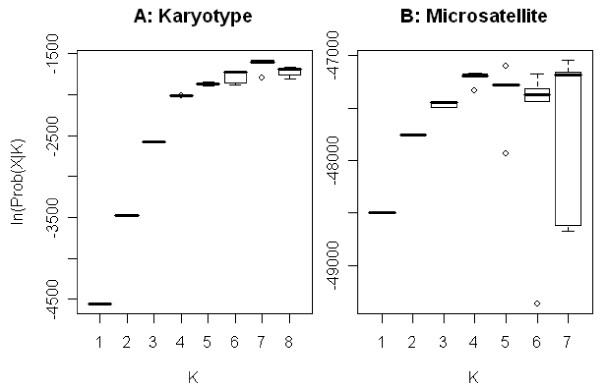
**Posterior probabilities of karyotype and microsatellite based Bayesian cluster analyses**. The boxplots were drawn after collecting posterior probabilities of 5 independent runs. The medians were drawn as thick black lines. Some boxes appear as a single line because the range of the posterior probabilities is too narrow to show as a box. Outliers are shown as open circle (○). A: Clustering of individuals into seven groups (K = 7) was the most probable solution for molecular form and karyotype data. B: Analysis of clustering of genotypes at 20 microsatellite loci yielded several outcomes with relatively high likelihood. Liklihoods for K = 4, K = 5 and K = 7 are similarly distributed (Wilcox twosample test, P > .05).

Finding the most succinct classification of karyotypes was attempted by employing a Bayesian clustering analysis, the results of which are depicted in Figures 2 and 3. Each vertical line in Figure 2 represents a single mosquito and different colours represent different clusters. The length of each colour represents the membership coefficient for that individual for each corresponding population. The membership coefficient of an individual can be interpreted as the probability of belonging to a population, or how much of its genome originated from a population. Thus, if an individual is indicated mostly in one colour, that individual likely originated from the corresponding population. If an individual is represented in two colours of equal length, that individual has half of its genome originated from one population and the other half from another. The sum of the membership coefficients for each individual is 1. Individuals were arranged such that the latitude of the collection sites increases from left to right on the bar plots in Figure 2. In some areas, the colours appear to be a solid block because the corresponding individuals have identical karyotypes. Karyotypes associated with each of the six populations are provided in Table 2. An additional character was added for molecular form. 'M', for M form, 'S', for S form.

**Table 2:** Sample table site names, locations, sample sizes and molecular form and 2La inversion composition

		**Molecular form & 2La karyotype**						
			
**site N**		**M +/+**	**M +/a**	**M a/a**	**S +/+**	**S +/a**	**S a/a**	**M/S hybrid**
***CAMEROON***								

Tiko	63	50	0	0	13	0	0	0

Mutengene	13	6	0	0	7	0	0	0

Foumbot	39	0	0	0	21	13	5	0

Ndop	37	0	0	0	12	19	6	0

Godola	40	0	0	0	0	3	37	0

***MALI***								

Pemperena	58	0	0	1	2	7	48	0

Kela	91	0	4	25	0	3	59	0

Banambani	96	0	0	3	0	25	68	0

Nara	117	1	2	113	0	0	1	0

Banikane	25	0	0	25	0	0	0	0

Dire	16	0	0	16	0	0	0	0

Total	595	57	6	183	55	70	224	0

N = Total number of *An. gambiae *samples for each collection site. M = M molecular form, S = S molecular form, '+' stands for standard arrangement for 2La inversion, 'a' for inverted arrangement, and '?' for unknown arrangement.

The analyses depicted in Figures 2A and 3A included molecular form and six chromosome inversion genotypes as criteria. A range of values for K was tested, which denotes the hypothetical number of populations. The solutions for K = 7 have the highest likelihood (Figure 3A).

The *Forest *M form was classified as a discrete population, fixed for the standard arrangement (M------) for all six inversions and carrying the M ribosomal allele (Table 2, shown in green in Figure 2). These are the same individuals identified as 'M 2L+/+' in Figure 1 (marked in green) mostly found in evergreen broadleaf forest regions in Cameroon. The observed lack of inversion polymorphism is consistent with previous observations by Wondji *et al*. [14,35]. Although some inversion polymorphism in M forms was observed in their study these were restricted to woody savanna areas (Tibati, Dschang) or to habitats at the border of evergreen forests and woody savannas. Two M form samples from Mali (one from Kela and the other from Banikane) had standard 2R arrangements. However, both were homozygous for 2La (2La a/a), consequently the Bayesian models predicted that these two samples belong to the Mopti-M form group rather than Forest M form (See Additional file 2 for genotype and corresponding membership coefficients).

**Figure 4:**
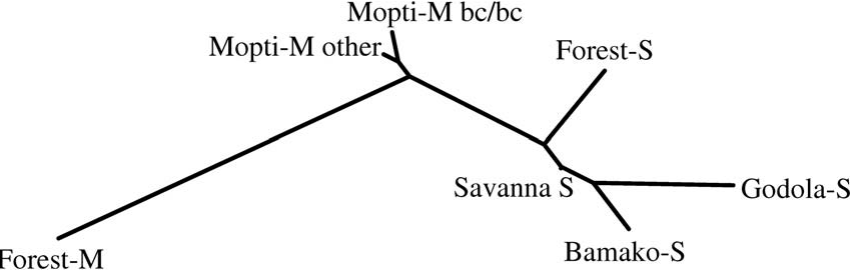
**Unrooted phylogenetic tree (neighbour joining) of the seven groups of *An. Gambiae *identified by Bayesian analysis (using *Structure *software), as illustrated in Figure 2A**. Tree is based on pair-wise F_ST _values derived from allele frequencies at 20 microsatellite loci on chromosomes 2 and 3. Distances between all branches are significant (see Table 3).

The Bayseian analysis based on molecular form and karyotype (Figure 2A) divided the *Mopti *chromosomal form into two groups: Mopti-M bc/bc (red) and Mopti-M "other" (orange). Significant deviation from Hardy-Weinberg equilibrium (HWE) was observed among M form populations at Nara (P = 0.0000 ± 0.0000) and Banikane (P = 0.00016 ± 0.00004). (Note that the M form population at Dire was excluded from HWE analysis because it is monomorphic for the bc karyotype (M2-22--; bc/bc)). The authors suspect that separation of the Mopti-M form reflects strong selection for the 2Rbc inversion in response to the arid conditions that exist at these sites. This observation is consistent with results reported by Touré *et al*. [3] who observed an increase in the frequency of 2Rb in M forms as precipitation decreases. However, this trend is not limited to M forms and a similar trend was also found in S forms (See Additional file 3). Overall, 2Rb inversion frequencies increased as precipitation decreases (Figure 4B)[5].

The *S *molecular form was divided into four groups, Forest-S (light blue), Savanna-S (blue), Bamako-S (purple), and Godola-S (yellow, Figure 2A). Forest-S forms were polymorphic for the 2La and 2Rb inversions, although inversion homozygotes for 2La or 2Rb were absent (Table 2). In contrast, inversion homozygotes for 2La and 2Rb were very common in the Savanna-S form (Table 2). The amount of gene flow between these two groups was relatively high as indicated by the presence of many individuals with mixed light blue and blue colors. This could be interpreted as active gene flow between the two groups, or less confidence in the clustering due to a lack of more discriminatory markers and/or the groups having relatively similar inversion frequencies.

Individuals homozygous for 2Rj, c, and u inversions were classified as the Bamako-S group (shown in purple in Figure 2A); this group represents the *Bamako *chromosomal form [5,9]. The 2Rb inversion was polymorphic within this group. Deviation from HWE were significant among S forms collected in Kela (P = 0.0000 ± 0.0000) and Banambani (P = 0.0000 ± 0.0000) as expected considering the S forms in these locations include both *Bamako *and *Savanna *chromosomal forms.

The samples collected in Godola were all of the S molecular form, but included unusual karyotypes that do not fit within the conventional definitions for *An. gambiae *chromosomal forms [5,9,36]. The Savanna form typically has the 2Rb inversion alone or in combination with the 2Rc and u inversions. At Godola, with the exception of a single individual, the 2Ru inversion was absent. The most common arrangement (82% of individuals) was 2Rbcd and 40% of the individuals sampled from this site were homozygous for the 2Rb, c and d inversions (Table 2). Deviation from HWE in the S-Godola population was significant (P = 0.0064 ± 0.0002), mainly due to 1 specimen with the S2-22-2 (bcu/bcu) genotype. Only six individuals (13%) had typical Savanna karyotypes. The 2Rd inversion is a very unusual arrangement both in Mali (freq. = 0.008) and Cameroon (freq. at sites other than Godola = 0.026). In Godola the 2Rd inversion occurred at a frequency of 0.544. Moreover, S form individuals carrying the 2Rbc combination (without 2Ru), typical arrangements for the Mopti chromosomal form (M molecular form), is noteworthy.

### Identification of discrete populations based on microsatellite genotypes

Genetic differentiation between identified groups was estimated using pair-wise F_ST _values based on 20 microsatellite markers on the second and third chromosomes. Results from this analysis indicate that the Forest-M group (M------) is the most genetically distant from all other groups. This result was consistent with the previous study by Slotman *et al*. [15]. The matrix of genetic distances is provided in Table 3. An unrooted tree calculated from the distance matrix using a neighbour-joining algorithm is provided in Figure 4. Genetic differentiation was low between the Mopti-M 2R bc/bc group and the "other" Mopti- M form (F_ST _= 0.0029, P = 0.016). Notice that genetic differentiation between the Savanna-S and Bamako-S groups were the smallest among the four S molecular form groups but still highly significant (F_ST _= 0.0056, P = 0.0000). These two forms are thought to mate assortatively and are considered to represent incipient species [5,37].

**Table 3:** Major karyotypes associated with each population identified in Bayesian clustering analyses.

**Clustering based on microsatellite genotype (K = 5)**	Forest-M	Forest-S	Savanna-S		Bamako-S	Mopti-M	
**Clustering based on karyotype (K = 7)**	Forest-M	Forest-S	Godola-S	Savanna-S	Bamako-S	Mopti-M bc/bc	Mopti-M other

**Color (Figure 2)**	Green	Light blue	Yellow	Blue	Purple	Red	Orange

**Molecular form**	M	S	S	S	S	M	M

**Karyotypes**		S ------(42)	S2-211-(15)	S2-2---(78)	S2212-2(52)		M2-11-1(50)
	M------(57)	S1-1---(21)	S2-222-(12)	S1-2---(27)	S22-2-2(39)	M2-22--(145)	M2-11--(39)
		S1-----(17)	S2-111-(3)	S2-1---(20)	S2222-2(15)		M2-21--(6)
		S--1---(12)					M2-----(2)

The six consecutive symbols describing karyotypes represent the genotype of 2La, 2Rj, 2Rb, 2Rc, 2Rd, and 2Ru listed in order. '-' represents standard (uninverted) homozygous arrangement, '1' heterozygote, '2' inverted homozygote.

The outcome of the Bayesian clustering analysis was compared based on karyotypes with those based on microsatellite genotypes (Figures 2B, C and 3B). Wilcox two-sample test indicates that K = 4 and K = 5 have similar likelihoods (P = 0.42). K = 5 generated a solution with the highest posterior probability Prob(X|K), where X is genotype data. Boxplots drawn after collecting posterior probabilities from 5 independent runs are presented in Figure 3B and resulting membership coefficients are plotted in Figure 2B (K = 4) and 2C (K = 5).

Under the assumption of K = 5 (Figure 2C), the Bayesian model identified the Forest-M form group (green) in Tiko and Mutengene, a Forest-S form (light blue) group, mostly concentrated in southern and central Cameroon, Bamako-S form group, Savanna-S group, and the Mopti-M form (red) group in Mali. The Savanna-S form group indicated in blue subsumes the Godola-S group identified based on karyotype (Figure 2A). Unlike karyotype clustering, the Mopti-M form remained as a single group based on microsatellite genotypes at both K = 4 and K = 5. This is reflected in the relatively low F_ST _values between the two groups (Table 3, Figure 4).

### Chromosome inversions, molecular forms and the environment

**Figure 5:**
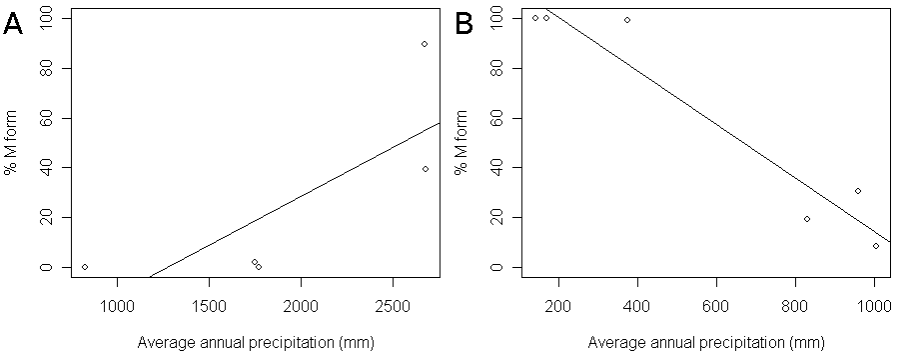
**Precipitation and molecular form**. Correlation between the proportion of M molecular forms and the average annual precipitation in (A) Cameroon and (B) Mali. Average annual precipitation was calculated using annual precipitation at multiple weather stations at or near collection sites (within a 15 km radius) and over multiple years. ○ = average annual precipitation at each site. Details of precipitation data are provided in Table S2. A. The proportion of the M forms increases as precipitation increases in Cameroon. B. The opposite trend was observed in Mali. These seemingly conflicting results illustrate that the Forest-M form and Mopti-M form differ with respect to habitat preference as well as being differentiated genetically. The solid line is a linear model (y~ax+b) of the data. (A) For the Cameroon data, a = 0.0395 ± 0.0184 (P = 0.121) and b = -50.5 ± 37.8 (P = 0.274). (B) For the Mali data, a = -0.108 ± 0.0150 (P = 0.00197) and b = 122 ± 10.3 (P = 0.000283).

As latitude increases in Cameroon the proportion of M form decreases whereas the inverse correlation was found in Mali. Generally, precipitation is higher at lower latitudes both in Mali and Cameroon (Table 4), but the amount of precipitation in southern Cameroon is 2–3 times higher than that in southern Mali. Average precipitation per collection site is summarized in Table 4 (detailed data are provided in Table S2). The correlation between the proportion of M molecular forms and annual precipitation in Cameroon and Mali is illustrated in Figure 5. The proportion of M form increases as precipitation increases in Cameroon (P = 0.00012). In contrast, the proportion of M form decreases as precipitation increases in Mali (P < 2 × 10^-16^). The dramatic difference in environmental preference between the Forest- M (Cameroon) and Mopti-M (Mali) is reflected in the genetic distance between the two illustrated in Table 3, Figure 4 and as described earlier by Slotman *et al*. [15].

**Table 4:** Estimation of pair-wise genetic divergence (F_ST_).

Label	Population	1	2	3	4	5	6
1	Forest M	-					

2	Forest S	0.04263***	-				

3	Godola S	0.04260***	0.02077***	-			

4	Savanna S	0.03410***	0.00733***	0.01126***	-		

5	Bamako S	0.04055***	0.01183***	0.01224***	0.00559***	-	

6	Mopti M other	0.02487***	0.01282***	0.02351***	0.01469***	0.02328***	-

7	Mopti-M bc/bc	0.02794***	0.01534***	0.02095***	0.01581***	0.02333***	0.00288*

Pair-wise F_ST _distance calculated based on 20 microsatellite loci among groups identified in the Bayesian clustering analysis (K = 7).

*P < 0.05, **P < 0.01, ***P < 0.001.

The International Geosphere-Biosphere Programme (IGBP) land cover scheme (Figure 1) identifies Tiko and Mutengene, where the Forest-M form is abundant, as an evergreen broadleaf forest region. On the other hand, the Mopti-M form is collected in the much drier savanna (Kela, Banambani), grassland (Nara), or open shrubland habitats (Dire, Banikane).

**Figure 6:**
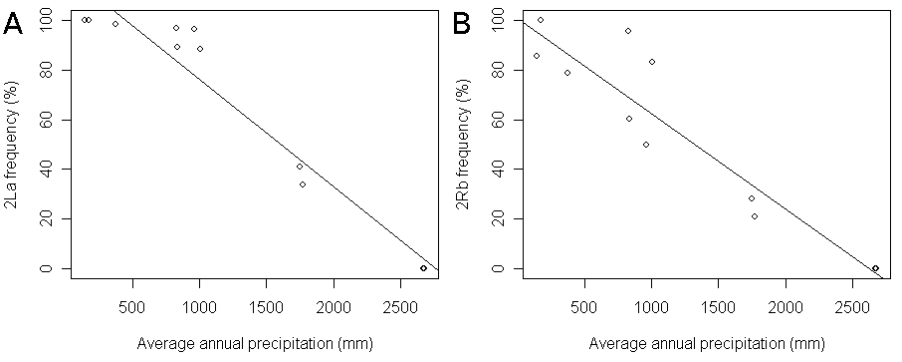
**Precipitation and chromosome inversion**. A: Inverse correlation between 2La inversion frequency and average annual precipitation. Average annual precipitation was calculated as for the data represented in Figure 5. ○ = average annual precipitation for each site. Details of precipitation data are provided in Additional file 1. The solid line is a linear model (y~ax+b) of data with coefficients of a = -0.0434 ± 0.00395 (P = 1.65 × 10^-6^) and b = 120 ± 5.85 (P = 7.39 × 10^-9^). B: Inverse correlation between 2Rb inversion frequency and annual precipitation. For a linear model, a= -0.0385 ± 0.00491 (P = 2.60 × 10^-5^) and b = 101 ± 7.25 (P = 2.16 × 10^-7^).

The correlation between the 2La inversion frequency and annual precipitation is shown in Figure 6A. The frequency of the 2La inversion decreased as the amount of precipitation increased (P < 2 × 10^-16^); a pattern consistent with previous studies in Africa [2,38]. As previously described, much fewer S form 2La homozygotes were observed in Cameroon and most of these were concentrated in Godola. All the study sites in Mali were much drier than those in Cameroon, except for Godola. This is reflected in the abundance of the 2La inversion in samples collected in Mali. Northern sites such as Nara and Banikane, located near the Sahara Desert, are particularly dry and the *Mopti *chromosomal form prevails at these sites. Some 2R standard arrangement were found in Mali, but all of them, except one, were 2La inversion homozygotes (M2-----); these were classified as the "other Mopti-M" group (orange in Figure 2A). The simple presence of the 2La inversion in M forms was sufficient to distinguish Forest-M from Mopti-M form in this case.

A similar trend was observed for the 2Rb inversion. The frequency of 2Rb increased as precipitation decreased, as reported by Coluzzi *et al*. [2]. Individuals homozygous for 2Rb (b/b) and 2La (a/a) had a high probability of being classified in the Savanna-S group rather than Forest-S group. Because of the inverse correlation between 2Rb and precipitation, division between the Forest-S group and Savanna-S or Godola-S groups is likely due to the skewed 2Rb frequency.

Clustering results based on microsatellite genotypes indicates that the Godola-S forms are relatively close to the Savanna-S and Bamako-S forms (Figure 2B and 2C, and Figure 4). The frequency of 2La and 2Rb in Godola were in line with the expectation based on precipitation. Genetic differentiation, based on microsatellite markers (Figure 2B and 2C), were also somewhat consistent with the association of land cover types and inversion polymorphism.

An unusual concentration of the 2Rd inversion in Godola resulted in the Bayesian analysis distinguishing it as a separate population (Figure 2A). Godola is located in an area surrounded by a mosaic of grasslands, permanent wetlands and croplands (Table 4). Permanent wetlands surround 19% of Godola, highest among our collection sites. A more detailed analysis of the effect of environmental parameters on the distribution of the 2Rd inversion will be necessary to gain an understanding of the relationship between the Godola-S form and other S forms.

The correlation between genetic markers and standard deviation of precipitation was also investigated. Areas with higher precipitation had greater variation in rainfall as well, as illustrated in Table 4. Thus the trend illustrated in Figures 5 and 6 remained the same between genetic markers and rainfall variation.

## Discussion

Classification of populations of *An. gambiae *s.s. into chromosomal forms using four paracentric chromosome inversions (2Rb, c, j and u) has been useful in identifying discrete sub-populations in Mali. Expanding this interpretation to include other areas in Africa has proven difficult because a significant proportion of karyotypes are ambiguous with respect to the classification scheme developed and used in Mali. Additional information, including molecular form, the 2La inversion and multi-locus microsatellite genotypes was employed in an attempt to obtain higher resolution of population structure in this species.

Seven groups were identified in the Bayesian analysis using molecular form, 2La, 2Rj, 2Rb, 2Rc, 2Rd, and 2Ru inversion genotypes as markers (Figure 2A). These seven groups are Forest-M, Forest-S, Godola-S, Savanna-S, Bamako, Mopti-M bc/bc, and other Mopti-M forms (Figure 2A). Each group showed distinctive inversion polymorphism, and each occupied a different land cover type (Figure 1). Bayesian analysis using microsatellite data for the most part supported the analysis based on chromosome inversions and molecular form, with some notable exceptions (Figures 2B and 2C). The microsatellite analyses at K = 4 and K = 5 resolved groups with roughly equivalent likelihoods (Figure 3B). Microsatellites failed to resolve the Godola-S group within the range of K values (K = [1,8]). At K = 4 the S form was divided into the Forest-S form and all other S forms. At K = 5 the other S form group was split into the Bamako and Savanna forms.

### The M form

The Bayesian analysis based on molecular form and karyotype divided M form populations into three discrete groups. The Forest M form was characterized as having the standard arrangement for all six major chromosomal inversions, namely 2La, 2Rj, 2Rb, 2Rc, 2Rd and 2Ru. The Mopti-M form was further divided into two groups, one that is fixed for the 2Rbc inverted arrangement and the other M forms with the 2La/a genotype. The Forest-M form was the most distinct (Figures 2 and 4). The high degree of genetic divergence between the Forest-M form and all the others was indicated not only by the 2R chromosome arrangement, but also at 20 microsatellite markers on the second and third chromosomes (Table 3, Figure 4). Moreover, the Forest-M form showed a strong association with very wet environments, distinguishing this form from the Mopti-M form, which is most abundant in much drier habitats (Figures 1 and 6, Table 4).

Despite occupying the same ecological zone and being identical with respect to karyotype (homosequential), the level of genetic differentiation (F_ST _= 0.04263, P = 0.0000) between the Forest-M and Forest-S forms was higher than among any of the other subspecific forms of *An. gambiae *s.s. (Table 3, Figure 4). The relationship between the Forest-M and Forest-S forms is clearly different from the relationship between the Mopti-M and Savanna-S forms in Mali. Hybridization between the Mopti-M and Savanna-S forms, although rare, has been frequently reported [8,39-41], whereas reproductive isolation between sympatric Forest-M and S forms appears to be complete [14,15,35]. These results demonstrate that reproductive isolation, and not ecological barriers, plays the major role in limiting gene flow between the Forest M and S forms in Cameroon and between the Mopti-M and Savanna-S forms in Mali.

The M forms were further sub-divided into two groups based on the Bayesian analysis of karyotype data (Mopti-M and "Other"-M, Figure 2A), while microsatellite data structured them as a single group (Figure 2B and 2C). The Mopti-M form is characterized as being fixed for the double homozygous b/c karyotype, whereas the "Other"-M form group carried the 2Rb and c inversions in other combinations or were standard karyotype. Both were fixed for the 2La inversion. Although genetic differentiation between the two groups as measured by pair-wise F_ST _is significant, the degree of divergence is relatively small and insufficient to classify them as two separate groups, that is as two distinct gene pools.

### The S form

The Bayesian clustering analysis based on molecular form and karyotype grouped S form populations into four group: Forest-S, Godola-S, Savanna-S and Bamako-S (Figure 2A). The Forest-S group includes individuals with the standard (non-inverted) karyotype and individuals carrying the 2La and/or 2Rb inversions. Forest-S 2Rb heterozygotes were located in woody savanna habitats (Ndop and Foumbot), while only Forest-S form with standard arrangement were collected from evergreen forest habitats (Mutengene and Tiko). This distribution may reflect selection for the 2Rb inversion in drier habitats. S form 2Rb homozygotes were classified as Godola-S form if the 2Rd inversion is also present, Savanna-S form if no other inversions occur on chromosome 2R, or Bamako-S if the 2Rj, 2Rc and 2Ru inversions occur together.

The Bamako-S form was not resolved by the analysis based on microsatellites with K = 4, but was resolved at K = 5. The Godola-S form however, was not resolved by the microsatellite data at either K = 4 or K = 5. The Godola-S group represents a distinctive group with respect to inversion polymorphism, notably a high frequency of the 2Rd inversion and 2Rb and c combinations not typically found in individuals within the S molecular form. However, the distinctive karyotypic polymorphism was not captured by the Bayesian analysis of microsatellite polymorphism.

Analysis of levels of genetic divergence, described with microsatellite-based F_ST _values (Table 3, Figure 4) suggest several relationships among the *An. gambiae *populations studied here. The major division distinguishes the Forest-M form from all others (Figure 4). S form populations form four groups.

Interestingly, the Bamako-S and Savanna-S forms are the least diverged of the four, although it is the Bamako chromosomal form that has received the most attention, including the suggestion that it represents a distinct species [37,42].

### Forces affecting the distribution of forms

Recently Yawson *et al*. [16] reported higher levels of genetic divergence between within-form populations from different environments relative to between-form populations from the same environments in Ghana and Burkina Faso. This led them to conclude that barriers to gene flow among populations are due more to ecological barriers than reproductive isolation between molecular forms. The two sites in Cameroon from which we described the Forest-M form occur within a region that is more or less contiguous with the mangrove strand sites in southern Ghana from which Yawson *et al*. [16] collected M forms for their study [43]. Furthermore, the 1 km resolution land-cover map of Africa by Mayaux *et al*. [43] indicates that land-cover in northern Ghana is similar to southern Mali. These results suggest that the M form collected by Yawson *et al*. [16] in northern Ghana represent the Mopti- M form while the M form they collected in the mangrove strand of southern Ghana are of the Forest-M form. It is, therefore, likely that what Yawson *et al*. [16] describe as intra-form comparisons of M form populations are in fact inter-form comparisons between Forest-M and Mopti-M forms. Overall, the results suggest that reproductive isolation among forms plays an important role in restricting gene flow among populations. Evidence laid out here does, however, support the idea that ecological barriers also play a significant role.

The correlations between 2La and 2Rb inversion frequencies and precipitation (Figure 6) were significant. In this study, precipitation is used as a proxy for different habitat type, rather than the causal factor for the distribution of 2La and 2Rb. Inversion polymorphisms in natural populations of *An. gambiae *s.s. are very complex and simple summary statistics, such as annual precipitation, are not sufficient to capture the diverse nature of the forces driving their distribution in nature. For example, the Godola collection has an unusual concentration of the 2Rd inversion which is difficult to explain by precipitation alone. Variation in rainfall was an obvious first environmental factor to consider, however describing this variable is not as trivial as it may seem. Rainfall is measured from 0 (meaning no rain) to some rain, and most of the regions included in this study have both rainy and dry seasons. Regions with high precipitation show greater variation in rainfall. Therefore, the correlation between genetic markers and the standard deviation of precipitation resulted in the same trend as the correlation with precipitation. Moreover, the standard deviation in rainfall is far greater than the mean (Table 4), illustrating the volatile nature of precipitation data. Examining the relationship between the distribution of genetic markers and rainfall in places like Tiko and Mutengene is further complicated because they have two rainy and two dry seasons. Other measures of rainfall variation, such as number of consecutive dry days or length of dry season can be explored. Development of summary statistics that may better reflect various rainfall patterns, as well as investigation of other environmental parameters are needed.

Microsatellites did detect population groups according to ecology and molecular forms. Division of Mopti-M, Savanna-S and Bamako-S groups are consistent with previous studies. Subdivision of the M form into Mopti-M and Forest-M forms has strong support in all of the Bayesian analyses presented here, as was subdivision of the S form into Savanna-S and Forest-S forms, although examination of Figures 2B and 2C suggest that the latter division is less clear.

## Conclusion

Overall this study confirms the Forest-M form as a distinct group, that lacks chromosome inversion polymorphism, is the most highly diverged of all the recognized subspecific forms of *An. gambiae *s.s. and is distinguished from other M molecular form populations by having a strong preference for environments with relatively high precipitation. The Forest-M form occurs in sympatry with the Forest- S form and reproductive isolation between the two appears to be complete, that is, no hybrids were observed. The relationship between the Forest-M form and Mopti-M form is less clear. It is clear that the two are relatively highly diverged, F_ST _= 0.02487 compared for example to the level of divergence between the Bamako and Savanna chromosomal forms in Mali, F_ST _= 0.00559. However, we lack good information as to whether the Forest-M and Mopti-M forms are reproductively isolated since we have not identified sites at which the two are sympatric.

When investigating associations between genetic markers and important phenotypes such as insecticide resistance and malaria parasite refractory features, disregard of existing population structure may lead to identification of spurious associations or may obfuscate true associations. The level of genetic differentiation and geographic distribution of the Forest-M form illustrates the potential danger of relying solely on molecular form for genotype/phenotype association studies.

## Competing interests

The authors declare that they have no competing interests.

## Authors' contributions

YL designed and performed experiments, analysed data and drafted the manuscript. CRM, AF, RDM, AGA, and AJC performed cytogenetic analysis. EF, SFT, AF, AJC, and GCL collected mosquito specimens. GCL designed experiments, discussed the results and implications and drafted the manuscript. All authors commented on the manuscript at all stages.

## Supplementary Material

Additional File 1 **Table S1**. Geographic information for each collection sites and annual precipitation recorded in stations near the collection sites. Click here for file

Additional File 2 **Table S2**. Karyotype and microsatellite genotype data of the mosquito samples used for this study. Also includes membership coefficients of K = 7 clustering based on molecular form and karyotype and membership coefficients of K = 5 clustering based on microsatellite. Click here for file

Additional File 3 **Table S3**. Abundance of 2Rb inversion in M forms in Mali and S forms in Cameroon. Click here for file
